# Identification of Genes Involved in the Responses of Tangor* (C. reticulata* ×* C. sinensis)* to Drought Stress

**DOI:** 10.1155/2017/8068725

**Published:** 2017-09-11

**Authors:** Jin-Ping Xiao, Lan-Lan Zhang, Hui-Qin Zhang, Li-Xiang Miao

**Affiliations:** ^1^Institute of Horticulture, Zhejiang Academy of Agricultural Sciences, Hangzhou 310021, China; ^2^School of Agriculture and Food Science, Zhejiang A & F University, Lin'an, Hangzhou 311300, China

## Abstract

Drought is the major abiotic stress with adverse effects on citrus, decreasing the agronomical yield and influencing the fruit quality. In this study, cDNA-amplified fragment length polymorphism (cDNA-AFLP) technique was used to investigate the transcriptional profile changes and identify drought-responsive genes in “Amakusa” tangor* (C. reticulata* ×* C. sinensis)*, a hybrid citrus sensitive to water stress. The 255 out of 6,245 transcript-derived fragments (TDFs) displayed altered expression patterns including (A) induction, (B) repression, (C) upregulation, and (D) downregulation. With BLAST search, the gene products of differentially expressed fragments (DEFs) could be classified into several categories: cellular processes, transcription, transport, metabolism, stress/stimuli response, and developmental processes. Downregulated genes were highly represented by photosynthesis and basic metabolism, while upregulated ones were enriched in genes that were involved in transcription regulation, defense, energy, and transport. Present result also revealed some transient and up- and then downregulated genes such as aquaporin protein and photosystem enzyme. Expression patterns of 17 TDFs among 18 homologous to function-known genes were confirmed by qRT-PCR analysis. The present results revealed potential mechanism of drought tolerance in fruit crop and also provided candidate genes for future experiments in citrus.

## 1. Introduction

Drought is the most severe and complex limiting factor for plant growth and crop production. The improved drought tolerance of crops is an urgent and strategic goal for plant biotechnology [1]. When exposed to drought stress, many plants have evolved mechanisms to cope with a restricted water supply. Plant adaptation to drought includes a series of morphological, physiological, and biochemical changes that are directly or indirectly under genetic control [2–5]. Finally, plant can modify growth and development to withstand drought environment through the changes in gene expression. The products of these genes not only play important roles in stress tolerance but also regulate the gene expression and signal transduction [6, 7].

Identification of drought-related genes is a key step for improving drought tolerance of crops by either breeding or genetic transformation. As a result of extensive studying in model plant* Arabidopsis*, great progress has been achieved in understanding the drought tolerance mechanism and various drought-responding genes involved in many different pathways were revealed and identified [8]. The products of these genes can be classified into functional proteins and regulatory proteins, mainly acting as regulation, signaling, and encoding proteins that support cellular adaptation to drought stress [9]. Significantly, many reports have investigated that the gene introduction can enhance drought tolerance in transgenic plants by regulating the biosynthesis of compatible solutes, such as amino acids, quaternary and other amines, and a variety of sugars and sugar alcohols [10–13]. Additionally, late embryogenesis abundant (LEA) proteins, heat shock proteins, and some transcription factors influencing the expression of a number of stress-related target genes have also been used to improve drought tolerance in transgenic plants [14, 15]. Thus, function analysis of stress-related genes is very important for understanding the molecular mechanisms of stress tolerance and improving the stress resistance of crops by gene manipulation [16, 17]. Although a great deal of drought-induced genes have been identified, most researches regarding plant drought tolerance have been focused on the model species* Arabidopsis* and the molecular basis of tolerance to water stress remains far from being completely understood in many other crops, especially in fruit crops.

Citrus is the most important fruit crop in the world. In China, plant region of citrus trees is mostly water deficit hilly area and hillside, which is frequently restricted by water deficiency in natural conditions, affecting both vegetative and reproductive processes. Thus, drought tolerance is one of the major traits that determine the ability of citrus to expand. Moreover, seasonal drought often occurs in summer and autumn. Almost every year, some region of China is hit by drought; for instance, severe drought reduced the citrus yields sharply in Zhejiang province during 2013. Thus, drought tolerance is necessary for citrus. Traditional breeding based on the drought tolerance has been always the important strategy in citrus, but it is much more time-consuming because of its complex reproductive biology, such as high heterozygosity, apomixis, polyembryony, cross- and self-incompatibility, and long juvenility. Genetic transformation may avoid genetic segregation and nowadays has been used as an effective tool to improve the target traits by introducing specific genes in citrus varieties [18]. Many efforts have been made to isolate and identify genes associated with stress response and several drought-responsive genes have been successfully obtained from citrus or its related genera [19–22]. Along with extensive use of genome-wide expression analysis technology, the abundance of EST data provides the basis for successfully obtaining drought-responsive genes from citrus by in silico cloning [20, 23, 24]. Though great progress has been made in gene isolation and functional characterization, more works on citrus in comparison with* Arabidopsis thaliana* and rice still need to be devoted to the study for gene functions identification and explanation of key signal pathways.

Currently, next generation sequencing technology (NGS) has been applied to provide global gene expression profiles of whole transcriptome. NGS expression studies are greatly facilitated by the availability of annotated genome sequence (reference or otherwise). As an alternative to NGS, the cDNA-AFLP technique is a widely adopted gene expression profiling method. This is especially true for crop plants which are awaiting whole genome- or whole transcriptome-sequence data. As a sensitive and efficient technology, the cDNA-AFLP has been widely used to seek and identify genes with changed expression under different environment conditions [25, 26]. This technique can avoid the possibility of different transcript-derived fragments (TDFs) arising from a single gene/cDNA by further improving [27, 28]. Moreover, its results correlate well with real-time PCR (Q-PCR) analysis [29]. In this study, we employed the cDNA-AFLP technique to investigate genome-wide transcriptome profiles of tangor plant under water deficit, identify the genes associated with drought stress response, and further validate the expression patterns for some candidate genes by quantitative real-time PCR (qRT-PCR). Present results will give us insight into the molecular mechanisms underlying drought tolerance in citrus.

## 2. Materials and Methods

### 2.1. Plant Material and Drought Treatments

Two-year-old “Amakusa” tangor* (C. reticulata* ×* C. sinensis)* seedlings, grafted on trifoliate orange [*Poncirus trifoliata* (L.) Raf.], were used for this investigation. The “Amakusa” tangor is sensitive to water deficiency and its drought tolerance is inferior to that of satsuma mandarin and Ponkan mandarin. Young uniform plants were selected and grown in plastic pots (33 cm diameter at the top, 25 cm diameter at the bottom, and 44 cm height) that were filled with 70% sand and 30% silt and clay. Seedlings were first established for 3 months with regular irrigation and fertilization under uniform greenhouse conditions (28°C, 16 h light, relative humidity oscillating between 50% and 70%) and then treated plants were severely deprived of water to the desired stress level. The control plants were watered daily throughout the stress period and kept the relative humidity oscillating between 50% and 70%. Leaf samples of treated plants were harvested at 9 a.m. on 3th day, 5th day, 8th day, and the 11th day. All samples were immediately frozen in liquid nitrogen after being harvested and stored at −80°C for subsequent RNA isolation.

### 2.2. Measurement of Leaf Water Status

Fully expanded leaves were detached at different time intervals (unstressed, 3, 5, 8, and 11 days after stress was initiated) from three representative plants of the three replications for relative water content (RWC) measurement described by White et al. [30]. RWC was calculated as follows: RWC  (%) = (FW − DW)/(TW − DW)100. Leaf water potential (*ψ*) was measured by using HR 33T (Wescor Inc., Logan, USA) in “dew point” mode, and measurements were carried out by means of leaf disc method.

### 2.3. RNA Isolation and cDNA Synthesis

Total RNA was extracted from approximately 4 g of frozen tissues using the Large-Scale Column Plant RNAout kit (Tiandz, Inc., Beijing, China). To eliminate the remaining genomic DNA, the RNA was treated with DNase I (Takara, Dalian, China) according to the manufacturer's instructions. The integrity of the total RNA was assessed by running a 2 *μ*l aliquot of the RNA sample on 1% formaldehyde denaturing agarose gel with ethidium bromide (EtBr). The concentration of RNA was estimated using a U-0080D spectrophotometer (Hitachi, Tokyo, Japan). Poly (A)^+^ RNA was isolated from 500 *μ*g of total RNA with PolyATtract mRNA Isolation Systems (Promega, Madison, WI, USA). Double-stranded (ds) cDNA was synthesized from poly (A)^+^ using the SMART™ cDNA Library Construction Kit (Clontech, USA) according to the manufacturer's instructions and then purified using the QIAquick PCR Purification Kit (QIAGEN, Germany).

### 2.4. cDNA-AFLP Analysis

To select the perfect primer pairs for this study, a total of 256 primer pairs that flowed from the permutation and combination of A, T, C, and G were synthesized. The polymorphism levels of all primers were evaluated by using of tangor under drought stress for 11 days and its normal control. Finally, 122 marker pairs with highly polymorphism and better amplification were picked out and performed in this study. The cDNA-AFLP procedure was performed as previously described by Bachem et al. [27] with some modifications. Approximately 1 *μ*g of ds cDNA was digested with 10 U of* Ase*I at 37°C for 4 h and then digested with 10 U of* Taq*I at 65°C for 4 h, followed by heat inactivation at 80°C for 20 min. The two steps of digestion were conducted in NEBuffer 3 (New England Biolabs, Inc., Beverly, MA) in a total volume of 50 *μ*l. The digested products were purified and ligated to adapters using 1 U of T4 ligase supplemented with T4 DNA ligase buffer (Takara, Dalian, China).

The 2.5 *μ*l diluted (1 : 10) ligated products was used as the cDNA template to perform preamplification in a 25 *μ*l reaction mixture containing 0.4 *μ*M of each primer (*Taq*I: 5′- GACGATGAGTCCTGACCGA -3′,* Ase*I: 5′- CTCGTAGACTGCGTACCTAAT -3′), 2.5 *μ*l 10x amplification buffer, and 1 U Taq DNA polymerase (Takara). The PCR was carried out using the following cycling parameters: 94°C for 5 min; 25 cycles of 94°C for 30 s, 56°C for 30 s, 72°C for 1 min, and 72°C for 10 min. For further selective amplification, the PCR solution included 1 *μ*l of 10-fold diluted preamplification products, 1.5 *μ*l 10x amplification buffer, 0.5 U Taq polymerase, and 0.4 *μ*M of each primer with selective nucleotides on the 3′ end (*Taq*I/*Ase*I: AA, AC, AG, AT, CA, CC, CG, CT, GA, GC, GG, GT, TA, TC, TG, TT) in 15 *μ*l total reaction volume. The PCR reaction was conducted following the program: 12 cycles: 94°C, 30 s; 65°C (−0.7°C/cycle), 30 s; 72°C, 1 min and 23 cycles: 94°C, 30 s; 56°C, 30 s; 72°C, 1 min; followed by a final extension step of 7 min at 72°C.

The PCR product was denatured at 95°C for 5 min after adding 10 *μ*l of 98% formamide loading buffer and then chilled on ice before loading onto gels. Four microliters of denatured PCR product was separated on 6% polyacrylamide gels containing 7 M urea at 75 W constant power for 2.5 h using a DNA sequencing gel system (Junyi, Beijing, China). The cDNA bands were visualized by silver staining according to the Silver Sequence™ DNA Sequencing System Technical Manual (Promega, Madison, WI, USA). The gel was naturally dried at room temperature and was then scanned using an EPSON V30 scanner. A representative picture of a sliver-stained cDNA-AFLP gel was presented in Figure S1 in Supplementary Material available online at https://doi.org/10.1155/2017/8068725. Gel images were analyzed and all visible AFLP fragments were scored in each lane. All of the oligonucleotides (adapters and primers) in the present study were commercially synthesized by Sangon (Shanghai, China). All of the amplification reactions of experiment were performed on a T1 Thermocycler (Biometra, Göttingen, Germany) according to the procedures described by JinPing et al. [31].

### 2.5. Cloning and Sequencing of Transcript-Derived Fragments (TDFs)

Differentially expressed cDNA bands, or transcript-derived fragments (TDFs), were excised from the polyacrylamide gel and used as templates to reamplify cDNA fragments. The reamplified products were purified with a 3S PCR Product Purification Kit (Biocolor BioScience & Technology Company, Shanghai, China) and cloned into the* pEASY*™-T1 vector (TransGen Biotech, Beijing, China) following the manufacturer's protocol; then the vector was transformed into competent* Escherichia coli* (TransGen Biotech) and finally sequenced using an ABI 3730 DNA analyzer (Applied Biosystems, Foster City, CA) by Sangon Biological Engineering Technology & Services Co., Ltd. (Shanghai, China).

### 2.6. Sequence Analysis and Functional Annotation

The homologs of TDFs sequences (with vector sequences trimmed off) were determined against the public nonredundant protein database (nr) at the National Center for Biotechnology Information (NCBI) (https://www.ncbi.nlm.nih.gov/BLAST/) using the BLAST algorithm [32]. The function-known genes from BLASTX search were compared to proteins from TAIR [33] and searched against MAtDB to assign functional classification based on the MIPS FunCat schema [34, 35].

### 2.7. qRT-PCR and Data Analysis

The gene expression was quantified using the 7300 Real-Time PCR System (Applied Biosystems, Foster City, CA, USA). The transcript-specific primers that were used in the qPCR (Table S1) were designed using an online Real-Time PCR Primer Design tool from GenScript Corporation (http://www.genscript.com/ssl-bin/app/primer). The citrus house-keeping gene *β*-*actin* was selected as the reference gene to normalize the amplification efficiency. For real-time PCR analysis, 1 *μ*l of 10-fold diluted cDNA samples was used in 25 *μ*l reactions containing 0.2 *μ*M each primer and 12.5 *μ*l of SYBR® Premix Ex Taq™ (Takara, Dalian, China). The PCR cycling conditions were 95°C for 30 s, 40 cycles at 95°C for 5 s, and 60°C for 31 s. To check the specificity of the amplified products, a dissociation curve was generated immediately after amplification, and, in the case of primer dimmers, a new set of primers was designed. Eight microliters of each sample was run on a 2% agarose gel and the images were visualized with ethidium bromide (EtBr). To determine the PCR efficiency of the target and reference genes, calibration curves were constructed using serial dilutions of the cDNA template.

The output data were analyzed by the instrument on-board SDS software (PE Applied Biosystems). All of the qPCR reactions were normalized using the C_T_ value of *β*-*actin* gene. The relative expression levels of the target genes were calculated with the formula 2^−ΔΔCT^ [36]. For each sample, triplicate quantitative assays were performed to ensure the reproducibility of the results, and the data were presented as means ± SE (*n* = 3).

## 3. Results

### 3.1. Changes in Leaf Relative Water Content

**Figure 1:**
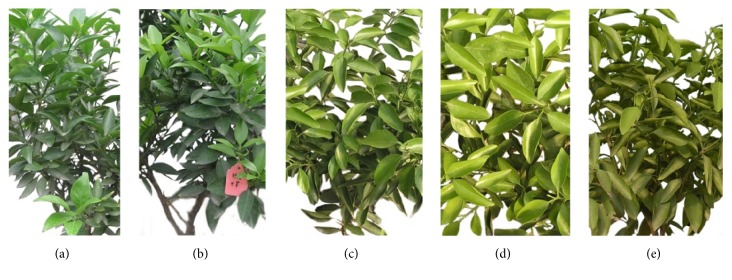
The detailed phenotype of leaf samples under drought stress. (a) The control sample; (b) leaf sample under drought stress for 3 days; (c) leaf sample under drought stress for 5 days; (d) leaf sample under drought stress for 8 days; (e) leaf sample under drought stress for 11 days.

The RWC was measured to investigate the water status during drought treatment and to determine the water deficit quantitatively in leaf tissues. During the entire 11 days, the control plants remained green and grew well, while the stressed plants were normal for the first 4 days after water withdrawing and then showed significant chlorosis and leaf rolling from the fifth day after dehydration. The detailed phenotypes under drought stress were presented in Figure 1.

**Figure 2:**
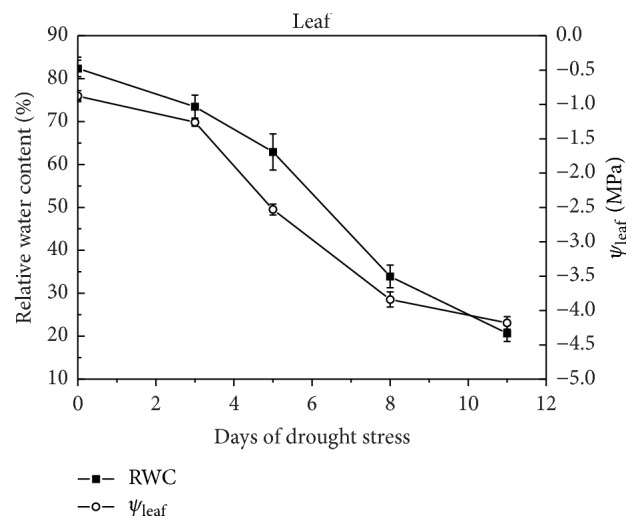
Changes of relative leaf water content and leaf water potential of “Amakusa” tangor during the drought treatment.

When drought started, the leaf RWC of the plants that were subjected to stress decreased gradually with drought time from 73% to 21% between the third day and the eleventh day of drought stress (Figure 2). In particular, the RWC of stressed plants experienced a rapid decline, from 62% to 33% from the fifth day to the eighth day of stress treatment. Changes in the leaf RWC under different drought conditions were also accompanied by changes in leaf water potential (*ψ*). Within 5 days between the 3th day and the 8th day, a decrease in*ψ* to approximately −3.84 MPa was observed (Figure 2). These results indicated that the stressed plants showed obvious changes in the phenotype and physiology. Thus, in the present experiment, the 11-day period was considered as an entire drought process including dynamic changes in drought responding.

### 3.2. Identification of Drought-Regulated Transcripts

To investigate the possibility of differential genes being induced by plant development or changes in the greenhouse environment during drought stress, the cDNA-AFLP procedure was conducted on the control plant using 3 randomly selected primer combinations (*Ase*I + AA/*Taq*I + AT,* Ase*I + CG/*Taq*I + GT and* Ase*I + TG/*Taq*I + CA). The results showed that no differentially expressed fragments were detected across the 11 days in the control plant (Figure S2).

**Figure 3:**
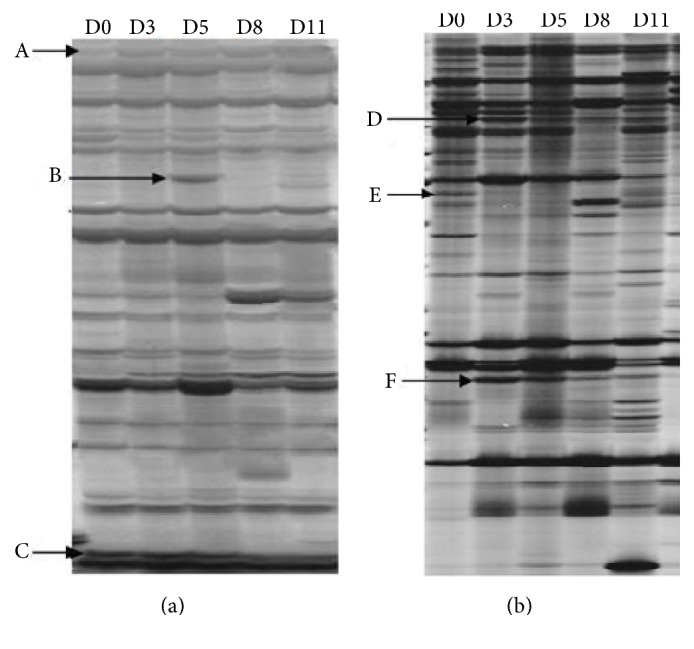
An example of cDNA-AFLP profile on stress-treated tangor. D0~D11: 0, 3, 5, 8, and 11 days after drought stress treatment, respectively; (a) and (b) cDNA-AFLP profile with AseI-CT/TaqI-GG and AseI-AT/TaqI-TC primer combination. The differentially expressed TDFs showed four expression patterns. A: upregulation; B, D, and F: induction; C: downregulation; E: repression. The TDFs of genes induced were present in drought stress but not in control. The TDFs of genes repressed were present in control but not in drought stress. The expression of upregulated and downregulated genes could be detected in both control and drought stress, with increased and decreased expression intensity under drought stress, respectively, when compared with control.

**Figure 4:**
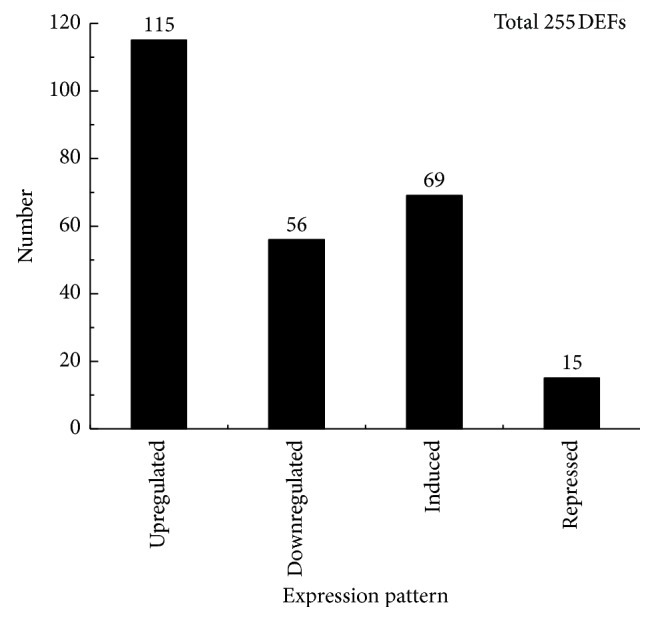
Distribution of the pattern of differentially expressed fragments revealed by cDNA-AFLP during drought treatment in “Amakusa” tangor.

The transcript expression of leaf samples across time intervals during the entire stress process was determined by cDNA-AFLP analysis. Approximately 6,245 TDFs were defined using 122 primer combinations with two selective nucleotides per primer. Each primer pair averagely produced 40 to 60 TDFs and these fragments varied from 50 to 800 bp in length, depending on their primer combinations and treatment period. For the statistics of differentially expressed TDFs, only the fragments that greater than 100 bp were considered. Polymorphic cDNA fragments were identified based on presence/absence (qualitative variants) and difference in expression intensity (quantitative variants). In general, quantitative variants were more predominant in number compared to qualitative variants.  Figure 3 showed an example of the expression patterns. A total of 255 TDFs with altered expression patterns were detected in comparison with the control, accounting for 4.1% of the obtained fragments. The expression pattern of 255 TDFs, including A: upregulated (115, 45%), B: downregulated (56, 22%), C: induced-expressed (69, 27%), and D: repressed-regulated (15, 6%) (A, B, C, and D represented genes that were upregulated, downregulated quantitatively, induced qualitatively, and repressed under drought stress in comparison with control, resp.) (Figure 4). The transcriptional profile appeared to be highly reproducible, as both the banding patterns and intensity of the amplified products were similar in the three biological replications for each treatment. All of the 255 TDFs were further analyzed, 229 of these were recovered, and 209 were sequenced.

### 3.3. Sequence Analysis and Functional Annotation

Within sequenced 209 TDFs, 175 produced reliable (>100 bp) sequences. The rest of 34 TDFs could not give unique sequence, probably due to being contaminated with comigrating fragments. Each sequence was conducted on a similarity search using the BLAST program against the GenBank nonredundant (nr) public sequence database.

**Figure 5:**
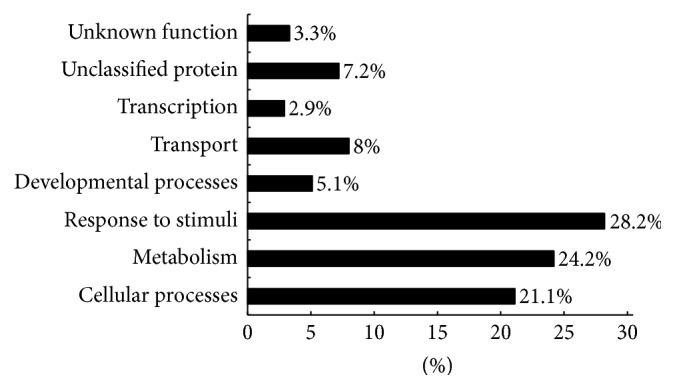
The functional distribution of differentially expressed fragments (DEFs) by modified MIPS based functional classification for “Amakusa” tangor. BLASTX comparisons to the predicted proteins from* Arabidopsis* were used to assign DEFs based on functional annotation after MIPS FunCat schema.

With the BLAST algorithm, 44% of the 175 sequences were found to have homology to genes with known function in the database (Table 1), whereas the majority of them belonged to either unknown proteins (24%) or zero matches (32%), indicating the enriched novel genes were included in citrus with drought stress. However, further study must be performed to identify the TDFs carrying unknown proteins or zero matches and explored how they can be applied against drought stress. The sequences with homology to functional genes from GenBank were individually searched against The Arabidopsis Information Resource (TAIR) and the Munich Information Center for Protein Sequences (MIPS) MAtDB databases. Sequence function was annotated according to the MIPS FunCat schema and of the 78 sequences returning a valid BLASTX hit, only 3.3% were found to encode function-unknown proteins. The encoding products of these genes could be subdivided into 12 functional categories and 7.2% of them were unclassified protein (Table 2). Most of the proteins were found in exceeding one functional category. Meanwhile, the number of the downregulation genes related to development and cellular biogenesis was nearly twice that of the upregulated. Additionally, as for transcription, energy, and transduction, the amounts of the upregulated and induced TDFs showed two times more than that of downregulated and repressed TDFs. These results indicated that drought may accelerate the degeneration of more development and cellular biogenesis and that gene transcription and stress defense can be enhanced by saving energy. These differentially expressed TDFs reflected the changing needs for sets of genes in defense, metabolism, and cellular processes during drought response in plants (Figure 5). A total of 18 TDFs were randomly picked for further expressional studies based on different expression patterns, including 6 induction, 1 repression, 9 upregulation, and 2 downregulation TDFs, all those TDFs were homology to function-known genes.

### 3.4. Validation of Differential Transcripts

The expression patterns of the 18 TDFs were analyzed by qRT-PCR to investigate the reliability of cDNA-AFLP analysis and to quantitatively assess the relative abundance of the transcripts. In most of the cases, qRT-PCR analyses showed the consistent results with cDNA-AFLP tests, except for TDF4 (being identified as upregulated during the stress), which gave significant variation, presumably on account of isolating a wrong fragment. In addition, the discrepancy of the results between qRT-PCR and cDNA-AFLP might also be due to their different sensitivity. However, further study should pay attention to TDF4 to explore its potential character in drought-related tangor.

**Figure 6:**
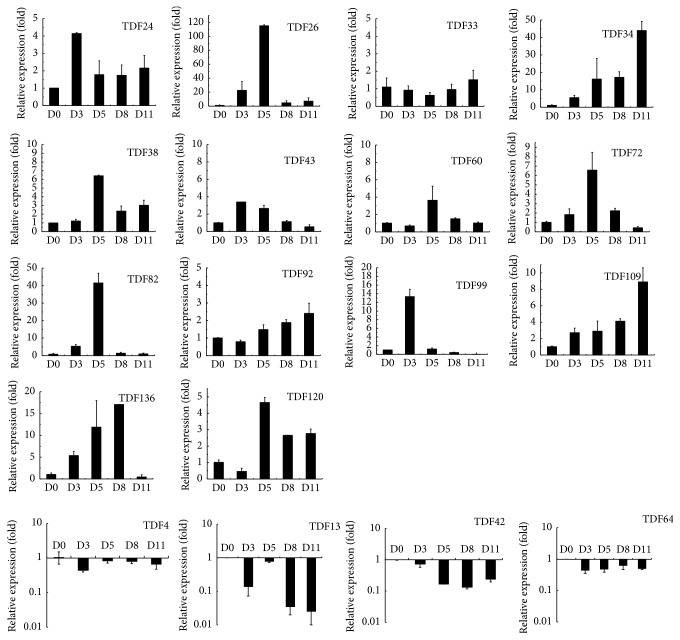
Quantitative real-time PCR (qRT-PCR) analyses of 18 differentially expressed transcripts for “Amakusa” tangor under drought treatments. Leaf tissues were sampled from plants on days 0, 3, 5, 8, and 11 after the start of water withdrawal. Three independent biological replications were performed. The relative expression level for stress-treated plants from 3 to 11 days was calculated as fold of control plants (0 day treatment) using the comparative ΔΔCT method. All data were normalized to *β*-actin. The mean expression value was calculated for every transcript-derived fragment (TDF) with three replications. (Induced genes: TDF24, TDF26, TDF38, TDF43, and TDF82; upregulated genes: TDF33, TDF34, TDF60, TDF72, TDF92, TDF99, TDF109, and TDF120, TDF136; downregulated genes: TDF13 and TDF42; repressed gene: TDF64; inconsistent gene: TDF4.)

Among the 14 genes that were upregulated or induced, the expression of 9 genes increased and peaked at 3 to 5 d after stress, while the expression of the other 5 genes peaked at 8 to 11 d after stress (Figure 6). The expression of 4 genes, TDF26 (encoding carboxypeptidase), TDF82 (encoding hypoxia responsive), TDF99 (encoding mitochondrial carrier), and TDF136 (encoding xyloglucan endotransglucosylase/hydrolase protein 9 precursor) rapidly increased, peaking from 3 to 8 d after drought and then sharply decreasing to the almost same level as the control. Moreover, qPCR analysis showed that the expression of only 4 TDFs continued to increase during stress, while the expression of the remaining 10 was up- and then downregulated. These results indicated that some genes might be triggered first by stress and play an active role during the early drought, thereafter being inactivated by continued or severe stress. The other 2 genes, cytochrome P450 (TDF13) and pyruvate kinase (TDF42), were continuously downregulated, while zinc finger protein (TDF64) was repressed after drought compared with normal plants. The expression of TDF13 in particular, a photosynthesis-related gene, showed significant downregulation at 3 d after stress, followed by a slight increase and then a rapid decrease to dozens of times the level at 11 d after treatment.

## 4. Discussion

Water deficit alters plant gene expression and leads to specific gene induction [37]. The identification and detailed analysis of a large number of candidate genes that are involved in drought tolerance may enable the elucidation of the molecular basis of drought resistance complexity. To fully uncover the TDFs existing on citrus with drought stress, 122 core marker pairs that were derived from 256 marker combinations were used. With the cDNA-AFLP technique, we detected 6,245 reproducible TDFs, demonstrating that the cDNA-AFLP technique is an efficient tool to study the genes that are involved in tolerance or resistance to environmental stress, the banding patterns of which are highly reproducible in cDNA-AFLP compared to other techniques, such as differential display [38]. Furthermore, each primer pair averagely produced 40 to 60 TDFs, indicating the efficiency and comprehensively of our selected markers. Although the present results showed minor differences in the expression data between cDNA-AFLP and qPCR due to the different detection sensitivities, these differences do not prevent a clear determination of expression patterns.

Previous studies have reported that many identified genes or transcripts are stress-inducible or upregulated, while the downregulated or repressed genes have been frequently underestimated [39, 40]. However, the expression profile analysis of our experiment found some transiently expressed and up- and then downregulated genes that could not be detected when only one or two time-point stresses were measured. Similar results have also been obtained in* Festuca mairei*, a xerophytic adapted grass [41]. The plant response to stress has a complicated regulation network underlying numerous physiological and biochemical changes. Various genes, including those that are downregulated or that undergo other types of regulation, are involved in drought response or even drought tolerance. In this experiment, four kinds of expression patterns were uncovered by the cDNA-AFLP analysis.

### 4.1. Drought-Inducible Genes

A large number of drought-induced genes with diverse functions have been identified by molecular and genomic analysis in a wide range of plant species, such as the dehydration responsive element-binding protein (DREB)/C-repeat binding factor (CBF) family [9, 42], and some of these genes via gene transfer have resulted in improved plant stress tolerance [10, 11]. Based on current knowledge, the functions of these genes mainly included directly protecting against environmental stresses and indirectly resisting by regulating gene expressions and signal transductions in the stress response.

In this research, 69 TDFs were identified as drought-induced gene fragments, and their encoded proteins included fructose-bisphosphate aldolase, cold acclimation WCOR413-like protein, multidrug resistance pump and DnaJ chaperone protein, which have been reported in association with stress response, the results were also validated by the previous studies [43–46]. The* PIP2* protein, an aquaporin that specializes in osmotic fluid transport [47], functions as a water channel to transport water through the plasma membrane and tonoplast to adjust the osmotic pressure under stress conditions [48, 49], exhibiting an inducible expression pattern during drought stress and peaking at expression level in the early stress period (3 days after water withdrawal), indicating that the expression of this gene might play a critical role in early drought defense. In addition, the products of these inducible genes include some regulatory proteins. The cleavage and polyadenylation specificity factor [50], cyclic nucleotide-gated ion channel [51] and auxin response factor [52], which are encoded by transcripts TDF21, TDF38 and TDF80, respectively, are involved in the regulation of signal transduction and stress-responsive gene expression and were also induced under drought, possibly governing the expression of stress-inducible genes either cooperatively or independently. Sedoheptulose-1, 7-bisphosphatase (SBPase), encoded by TDF3, is a key enzyme of the calvin cycle governing the photosynthetic rates and levels of Suc and starch that accumulate during the photoperiod [53]. Interestingly, induction-pattern SBPase showed up-then-down regulated expression during the whole period of drought treatment, indicating that the activity of SBPase may be stimulated by minor drought but inhibited by severe drought. Different cysteine proteases (TDF221) have been characterized in plants, where they participate in various proteolysis activities. Moreover, cysteine proteases have been reported to function in relation to senescence and programmed cell death [54], from our study, drought changed the expression of the genes encoding cysteine proteases, which might indicated that drought stress was mediated by senescence and programmed cell death.

### 4.2. Drought-Repressed Genes

Drought-repressed genes, as well as drought-induced genes, also have important functions for the survival and eventual development of the plant during water stress [37]. In this experiment, the number of expression-repressed genes with high homology to function genes was far less than that of inducible genes, suggesting that the functions of most drought-repressible genes remain unknown and require more study to increase our knowledge of the gene network governing drought tolerance. Iron-sulfur (Fe-S) clusters have been found in the mitochondrial electron transport chain [55] and function as important regulatory sensors that respond to oxidative stress and intracellular iron levels [56]. TDF115 encodes an iron-sulfur cluster assembly scaffold protein, a NifU-like protein that helps to assemble or repair iron-sulfur (Fe-S) clusters by acting as a scaffold, the repressed expression of which might be due to the reduction of photosynthesis. Recent research proved that succinate dehydrogenase is a direct source of reactive oxygen species (ROS) in plant mitochondria and regulate plant development and stress responses, inhibition of which can increase resistance of plants to abiotic stress [57]. Our results found that TDF176 encoded succinate dehydrogenase, the results revealed that ROS was involved in the drought stress of citrus. Recently, evidence has indicated that chromatin remodeling is involved in abiotic stress responses and stress tolerance [58], our study revealed that TDF 247 encoded chromatin remodeling, indicating its potential role in drought stress. However, the mechanism of the chromatin remodeling genes that are involved in drought defense is still not clear and requires more study.

The 26S proteasome subunit was presented in both the repressed and upregulated groups in this study. Similar results have also been found in* Arabidopsis* and rice [8, 59]. It is impossible to estimate the role and importance of this subunit in tolerance or sensitivity only based on controllable experiment conditions. However, these differentially expressed TDFs provide clues regarding the genes that are differentially expressed with a reference database for later comparison with data from natural field drought conditions.

### 4.3. Genes Upregulated to Drought Stress

Present results showed that the upregulated express pattern contained the most genes, with high homology to function proteins. Some proteins for transcription and translation regulation were upregulated, such as zinc finger protein, MYB transcription factor, translation initiation factor, and global transcription factor, indicating that the activity of partial stress-responsive genes is increased by these factors for positive stress defense. Small heat shock proteins (sHsps) encoded by TDF213 and TDF251, a group of proteins with a molecular mass of 15 to 42 kDa, can be induced by environmental stresses and developmental stimuli and function as molecular chaperones that bind to partially folded or denatured substrate proteins, thereby preventing irreversible aggregation or promoting correct substrate folding [60]. Positive relationships between sHsp levels and tolerance to desiccation have also been reported [61]. TDF120 has high homology to a beta-amylase, which is known for its function in the breakdown of starch to produce maltose. Maltose has the ability to protect proteins, membranes, and the photosynthetic electron transport chain. Beta-Amylase induction and the resultant maltose accumulation may function as a compatible solute-stabilizing factor in the chloroplast stroma in response to acute temperature stress [62], the upregulation of which can affect osmotic protection. Brassinosteroid insensitive 1 (BRI1), acting as an essential component of the BR receptor, participates in stress response signaling through interactions with ligands and proteins that are involved in plant defense responses [63]. TDF72 encodes a BRI1-associated receptor kinase 1 (BAK1), a second LRR RLK that interacts with BRI1* in vitro*, and may play an important role in BR signal transduction [64, 65]. This result suggests that the production of brassinosteroid could start to increase the tolerance of plants imposed by drought stress.

Additionally, some genes encoding proteins that are involved in energy and metabolism appeared to be upregulated, including 2, 4-dienoyl-CoA reductase, triacylglycerol lipase, pyridoxal biosynthesis protein (PDX1), xyloglucan endotransglucosylase/hydrolase protein 9 precursor, hydrolyzing O-glycosyl compounds, and anthranilate phosphoribosyltransferase. The xyloglucan endotransglucosylase/hydrolases (XTHs) are thought to play a role in cell wall restructuring, cell expansion, and, therefore, root growth [66]. XTHs may be recruited to alter tissue tensile strength or flexibility, enabling adaptation to mechanically stressful environments [67]. Pyridoxal biosynthesis protein (PDX1) is involved in the biosynthesis of vitamin B6, playing a role in the stress tolerance and photoprotection of plants; the loss of this protein leads to shorter, pyridoxine-dependent root growth and hypersensitivity towards oxidative stress [68, 69]. In addition, 3 genes encoding transport-category proteins (cationic amino acid transporter, mitochondrial carrier protein, and importin alpha) also showed upregulation, and the expression alteration of these genes can drive some physiological adaptation in plants by transferring energy and protein molecules, contributing to drought tolerance. In this study, an unknown gene containing WD40 domains was upregulated under drought. WD40 repeated proteins are key regulators of plant-specific developmental events [70–72], and a correlation between WD40 proteins and salt stress in crops has been reported [73, 74]. Further study aiming to explore the function of WD40 is necessary for the utilization on drought stress. According to the observations and results, our study indicated that the upregulated genes that mediated the drought stress might be through preventing irreversible aggregation, affecting osmotic protection, and interacting with ligands and proteins as well as energy and metabolism transformation.

### 4.4. Genes Downregulated in Response to Drought Stress

The identification and functional analysis of downregulated genes related to drought stress will improve our knowledge of the molecular mechanism controlling the drought resistance of plants. A total of 18 differentially expressed TDFs showed significant homology with previously identified proteins (Table 2). The number of downregulated genes was almost the same as that of induced genes (21), suggesting that downregulated genes in response to drought stress also play important and special roles in drought tolerance.

Downregulated genes were involved in a number of basic metabolic or biosynthetic functions and systemic development or growth, such as energy (hydroxyphenylpyruvate reductase), oligopeptide synthesis (ATP binding protein), amino acid metabolism (thiamin biosynthetic enzyme), carbohydrate metabolism (carbohydrate kinase family), and cell division (ATP-dependent RNA helicase). In addition, several proteins for transcription and signal transduction were also downregulated, including DNA-binding protein, big map kinase, and zinc finger protein. Some TDFs showing high similarity with photosynthesis-related genes were downregulated by drought, including cytochrome P450 monooxygenase, cytochrome P450, chloroplast chlorophyll a/b-binding protein, and photosystem I P700 chlorophyll a apoprotein A2. Photosynthesis depression seems to switch another carbohydrate utilization pathway, leading to the production of valuable stress tolerance molecules [75]. Altogether, it seems that the downregulation of photosynthesis-related genes possibly contributes to reduced photooxidation stress in tangor.

Pyruvate kinase (PK) is a key regulatory enzyme that catalyzes a rate-limiting step of glycolysis and has been reported to participate in the plant defense signaling transduction pathway [76]. The expression of the PK gene was induced during an incompatible interaction in hot pepper but downregulated during drought stress in this study, and the obvious difference suggests that pyruvate kinase might play different roles in biotic and abiotic stresses.

## 5. Conclusion

In summary, we successfully used the cDNA-AFLP technique to determine gene expression patterns in response to the drought stress of “Amakusa” tangor. A total of 255 TDFs with altered patterns of gene expression during stress were identified. A sequence analysis of the 175 TDFs identified genes that were involved in various molecular events during drought stress. These results revealed a variety of drought-responsive genes that related to drought stress of tangor at a comprehensive molecular regulation level. These genes mainly governed the function of cellular biogenesis, metabolism and cellular processes, and energy transportation, which provided insight into the response of citrus to drought tolerance and provide candidate genes for future function analysis. This study also discovered some novel genes whose functions remain unclear, indicating that tangor might apply a currently unknown defense mechanism against water deficit stress. The TDFs obtained in this study can provide a guidance for further exploration of drought stress related genes, which may lead to a comprehensive understanding of the molecular basis of citrus drought tolerance and will be helpful for the organization of genes that are involved in drought tolerance and crop improvement.

## Supplementary Material

Table S1: 18 TDFs and their primers for qRT-PCR analysis. Figure S1: An example showing transcript profiles at different water deficit treatment stages. Figure S2: cDNA-AFLP profile on control plants during the drought stress treatment.

## Figures and Tables

**Table 1:** Functional annotation and size of the differentially expressed fragments (DEFs) under drought stress in tangor.

TDF	Accession number	Sequence homology	Organism	*E* value	Size (bp)
*Transcripts indicating induced expression genes*					

3	GW811259	Sedoheptulose-1,7-bisphosphatase	*Cucumis sativus*	6*e* − 41	270
4	GW811260	Phosphatase 2C protein	*Eucalyptus grandis*	3*e* − 44	200
15	GW811265	rRNA intron-encoded homing endonuclease	*Oryza sativa*	1*e* − 15	176
21	GW811270	Putative cleavage and polyadenylation specificity factor 73 kDa subunit	*Ricinus communis*	3*e* − 18	159
24	GW811273	Putative fructose-bisphosphate aldolase	*Ricinus communis*	7*e* − 21	186
26	GW811275	D-Alanyl-d-alanine carboxypeptidase	*Ricinus communis*	5*e* − 18	205
29	GW811278	Putative DNA binding protein	*Ricinus communis*	1*e* − 09	151
38	GW811282	Putative cyclic nucleotide-gated ion channel	*Ricinus communis*	2*e* − 29	219
43	GW811285	Putative dihydrolipoamide dehydrogenase	*Ricinus communis*	7*e* − 47	317
80	GW811311	Auxin response factor 3	*Cucumis sativus*	1*e* − 16	203
82	GW811312	Hypoxia-responsive family protein	*Citrus sinensis*	8*e* − 09	202
132	GW811328	Putative multidrug resistance pump	*Ricinus communis*	2*e* − 06	224
137	HO008480	Cold acclimation WCOR413-like protein	*Poncirus trifoliata*	8*e* − 10	201
160	HO008493	Self-incompatibility (S-) locus region	*Ipomoea trifida*	8*e* − 06	268
171	HO008503	Chaperone protein dnaJ 15	*Citrus sinensis*	2*e* − 25	172
215	HO008535	Leucine-rich repeat family protein	*Arabidopsis thaliana*	2*e* − 21	332
221	HO008541	Cysteine protease	*Citrus sinensis*	8*e* − 24	171
223	HO008543	LSH6 (LIGHT SENSITIVE HYPOCOTYLS 6)	*Arabidopsis thaliana*	7*e* − 68	480
232	HO008550	Al-induced protein	*Gossypium hirsutum*	3*e* − 93	712
242	HO008555	Putative protein phosphatase 2c	*Ricinus communis*	2*e* − 22	204
250	HO008562	putative aquaporin PIP2.2	*Ricinus communis*	9*e* − 44	339

*Transcripts indicating repressed expression genes*					

18	GW811267	18S rRNA gene	*Symplocos paniculata*	5*e* − 25	171
64	GW811302	Putative zinc finger protein	*Ricinus communis*	2*e* − 22	237
115	GW811320	Iron-sulfur cluster assembly scaffold protein	*Chlamydomonas reinhardtii*	3*e* − 06	178
176	HO008507	Putative succinate dehydrogenase	*Ricinus communis*	1*e* − 41	315
187	HO008516	26S proteasome regulatory subunit	*Gossypium hirsutum*	3*e* − 22	281
247	HO008559	Chromatin remodeling complex subunit	*Populus trichocarpa*	1*e* − 18	388

*Transcripts indicating upregulated genes*					

25	GW811274	Putative 2,4-dienoyl-CoA reductase	*Ricinus communis*	7*e* − 13	149
33	GW811279	Putative ATP binding protein	*Ricinus communis*	1*e* − 06	260
34	GW811280	26S proteasome subunit RPN2b	*Arabidopsis thaliana*	6*e* − 27	217
57	GW811295	Putative eukaryotic translation initiation factor 3 subunit	*Ricinus communis*	4*e* − 17	213
60	GW811298	MutT-like protein	*Cucumis sativus*	1*e* − 10	146
65	GW811303	Putative triacylglycerol lipase	*Ricinus communis*	1*e* − 18	409
66	GW811304	Cationic amino acid transporter	*Populus trichocarpa*	4*e* − 26	361
71	GW811306	Uncharacterized protein	*Citrus sinensis*	2*e* − 52	161
72	GW811307	BRASSINOSTEROID INSENSITIVE 1-associated receptor kinase 1 precursor	*Ricinus communis*	7*e* − 13	158
92	GW811314	Hypoxia-responsive family protein	*Citrus sinensis*	8*e* − 09	202
99	GW811315	Putative mitochondrial carrier protein	*Arabidopsis thaliana*	4*e* − 07	143
109	GW811317	Similar to Probable pyridoxal biosynthesis protein PDX1	*Vitis vinifera*	7*e* − 18	213
110	GW811318	Ethylene-inducible protein (ER1)	*Hevea brasiliensis*	5*e* − 21	213
120	GW811325	Putative beta-amylase	*Ricinus communis*	1*e* − 08	132
136	HO008479	Putative xyloglucan endotransglucosylase/hydrolase protein 9 precursor	*Ricinus communis*	1*e* − 20	202
147	HO008487	Importin alpha	*Citrus sinensis*	9*e* − 65	644
148	HO008488	Hydrolase, hydrolyzing O-glycosyl compounds	*Ricinus communis*	6*e* − 102	612
149	HO008489	Glycine-rich RNA-binding protein	*Citrus unshiu*	2*e* − 30	567
159	HO008492	putative 26S proteasome subunit RPN2a	*Capsicum chinense*	2*e* − 23	295
161	HO008494	Transducin family protein/WD-40 repeat family protein	*Arabidopsis thaliana*	2*e* − 04	250
165	HO008497	Global transcription factor group	*Populus trichocarpa*	2*e* − 06	209
172	HO008504	Serine-type endopeptidase	*Arabidopsis thaliana*	5*e* − 06	163
179	HO008510	Putative natural resistance-associated macrophage protein	*Ricinus communis*	3*e* − 34	270
185	HO008514	Putative 60S ribosomal protein L31	*Ricinus communis*	4*e* − 35	449
197	HO008524	Putative heat shock protein binding protein	*Ricinus communis*	1*e* − 13	230
200	HO008526	Putative transcription factor IIA large subunit	*Arabidopsis thaliana*	9*e* − 13	284
201	HO008527	Anthranilate phosphoribosyltransferase	*Populus trichocarpa*	5*e* − 05	280
206	HO008529	PHD finger family protein	*Arabidopsis thaliana*	4*e* − 05	155
213	HO008534	Small heat shock protein	*Solanum lycopersicum*	2*e* − 22	301
224	HO008544	Putative ring finger protein	*Ricinus communis*	9*e* − 32	442
225	HO008545	MYB transcription factor MYB180	*Glycine max*	3*e* − 42	375
227	HO008546	Dehydrin	*Citrus sinensis*	2*e* − 10	312
229	HO008548	Putative protein COBRA precursor	*Ricinus communis*	1*e* − 22	233
234	HO008551	40S ribosomal protein S2 (RPS2D)	*Arabidopsis thaliana*	2*e* − 52	367
251	HO008563	Small heat shock protein	*Solanum lycopersicum*	1*e* − 22	298

*Transcripts indicating downregulated genes*					

10	GW811262	DNA-binding protein-related	*Arabidopsis thaliana*	4*e* − 07	140
13	GW811264	Cytochrome P450 monooxygenase CYP90A15	*Glycine max*	1*e* − 08	145
42	GW811284	Putative pyruvate kinase	*Ricinus communis*	5*e* − 10	336
62	GW811300	Miraculin-like protein 2	*Citrus jambhiri*	3*e* − 06	174
116	GW811321	18S rRNA gene	*Symplocos paniculata*	5*e* − 25	171
126	GW811326	Hydroxyphenylpyruvate reductase	*Salvia officinalis*	1*e* − 15	261
146	HO008486	Putative beta-amylase	*Ricinus communis*	8*e* − 112	664
166	HO008498	Cytochrome P450	*Citrus sinensis*	9*e* − 29	204
177	HO008508	Putative ATP-dependent RNA helicase	*Ricinus communis*	8*e* − 38	256
193	HO008520	Putative mannose-P-dolichol utilization defect 1 protein	*Ricinus communis*	3*e* − 27	369
222	HO008542	Big map kinase/bmk	*Ricinus communis*	3*e* − 122	702
243	HO008556	Carbohydrate kinase family	*Arabidopsis thaliana*	6*e* − 25	202
245	HO008558	Chloroplast chlorophyll a/b-binding protein	*Agave tequilana*	1*e* − 84	487
248	HO008560	Photosystem I P700 chlorophyll a apoprotein A2	*Anomochloa arantoidea*	4*e* − 14	369
249	HO008561	Thiamin biosynthetic enzyme	*Platanus x acerifolia*	2*e* − 18	351
254	HO008565	ATP binding protein	*Ricinus communis*	3*e* − 16	265

**Table 2:** Distribution of the differentially expressed fragments (DEFs) during drought stress by functional categories.

Functional category	%	Upregulated (%)	Downregulated (%)	Induced (%)	Repressed (%)
Cellular processes	21.1	10.2	12.5	9.7	6.1
Development	3.9	2.5	5.8	4.4	10.7
Cell organization and biogenesis	1.2	3.7	6.9	2.5	15.2
Metabolic processes	19.2	14.8	17.7	21.2	19.8
Protein metabolism	4.3	5.2	6.7	1.4	0.0
DNA or RNA metabolism	0.7	0.0	1.4	0.0	2.3
Stress response	14.1	17.6	11.4	18.1	18.7
Response to abiotic or biotic stimulus	13.0	9.8	7.1	10.7	5.3
Signal transduction	1.1	3.2	2.2	0.6	0.0
Transport	6.2	9.8	7.8	15.8	6.7
Electron transport or energy	1.8	4.1	3.3	5.6	0.0
Transcription	2.9	8.6	0.0	1.8	0.0
Unclassified protein	7.2	4.4	6.9	3.3	4.5
Unknown function	3.3	6.1	10.6	4.9	7.4

Classification was performed for 119 DEFs with statistical similarity to GenBank plant protein sequence (*E* values lower than 1.00*E* − 05) by BLAST search. The functional category was assigned based on function classification criteria in the website of Munich Information Center for Protein Sequences (MIPS) (https://www.helmholtz-muenchen.de/ibis/).
