# Supplementary material for: Identification of Genes Involved in the Responses of Tangor (C. reticulata × C. sinensis) to Drought Stress
Source: Biomed Res Int. 2017 Sep 11;2017:8068725. doi: 10.1155/2017/8068725 (PMC5612316; doi:10.1155/2017/8068725)
Supplement: Supplementary file 1 — Table S1: 18 TDFs and their primers for qRT-PCR analysis. Figure S1: An example showing transcript profiles at different water deficit treatment stages. Figure S2: cDNA-AFLP profile on control plants during the drought stress treatment. [file 8068725.f1.pdf]

Supporting Information

Table S1 Primers of selected genes used for qRT-PCR analysis and their amplification size.

**Name**	**Predict coding protein**	**Primer sequence (5'-3')**	**Product length (bp)**
TDF4	Phosphatase 2C	F: ATACCGAGACCATGAACAAG<br>R: AGTGGATGAAGAGGACAAA	169
TDF13	Cytochrome P450 monooxygenase	F: TACCTCTTCTGCGTTCTTGT<br>R: CTGCGTACCTAATACCAGTAAA	109
TDF24	fructose-bisphosphate aldolase	F: AATACATCTCTGGTGCCATC<br>R: AGTCCTGACCGACTTTGATA	123
TDF26	D-alanyl-d-alanine carboxypeptidase	F: TGGAGGAAATGTTAAACGAG<br>R: CCTAATCGCCACAATATCAT	179
TDF33	ATP binding protein	F: TACGGTGAAACCAATACTCC<br>R: CGAAATCAGACATTCCTCTC	157
TDF34	26S proteasome subunit RPN2b	F: GCAGTAGGTATCTCCTGTGC<br>R: GACTGCGTACCTAATCTGGA	144
TDF38	cyclic nucleotide-gated ion channel	F: CTTGAAATTTGTAGCCTCTCA<br>R: TGCTTGTATGAAACATGCTG	112
TDF42	pyruvate kinase	F: GCGTCAAGAATCTACTCACC<br>R: TAGAGTCTTGGGCTCTCAAA	180
TDF43	dihydrolipoamide dehydrogenase	F: TCTTAGCTGGTAAACATGGC<br>R: CCAAGTTCCTTTACCTGCTC	112
TDF60	MutT-like protein	F: GGATCTTGAAGTTCTCGTCA<br>R: CTCGTAAAGCTGCTTCTTGT	104
TDF464	zinc finger protein	F: GTACCAAGGACTTCTTCGC<br>R: TGGGAAATCTTCAACAAATC	113
TDF72	BAK1 precursor	F: CAACATGGACCTAACATCCT<br>R: AATGGGTATGAGGAGCAAAT	105
TDF82	Hypoxia-responsive family protein	F: CCTAATGCCTCCTCAGATAA<br>R: CGCACACAAGGATTAAATAAC	111
TDF92	Hypoxia-responsive family protein	F: CGCACACAAGGATTAAATAAC<br>R: CCTAATGCCTCCTCAGATAA	111
TDF99	mitochondrial carrier	F: ATGAGTCCTGACCGAATACA<br>R: CAACTGTTTCCATCCTTGAT	112
TDF109	pyridoxal biosynthesis	F: GGTGTGATGGAGTGTTTGT<br>R: GTACCTAATGCCCACCATAG	158
TDF115	Iron-sulfur cluster assembly scaffold	F: CTGACCGACAAGAGCGAT<br>R: TGCCACTCAAACAGATACAG	156
TDF120	beta-amylase	F: CTGACCGACACCATGTTTAT<br>R: GAATGGACCTTTCAGATGAG	104
TDF126	Hydroxyphenylpyruvate	F: GCGTACCTAATCGTGAAGTC	168

	reductase	R: GCTGTGCTTTCTAAATGGTT	
TDF136	XTH protein 9 precursor	F: GGGTACTGGGTATCTCGATT<br>R: GGGAGGCAAATAATTATCAA	116
Reference	Citrus actin	F: GGAGTTCATTGTAGAAGGTG<br>R: GGCGGTGTTCCCCAGTAT	200

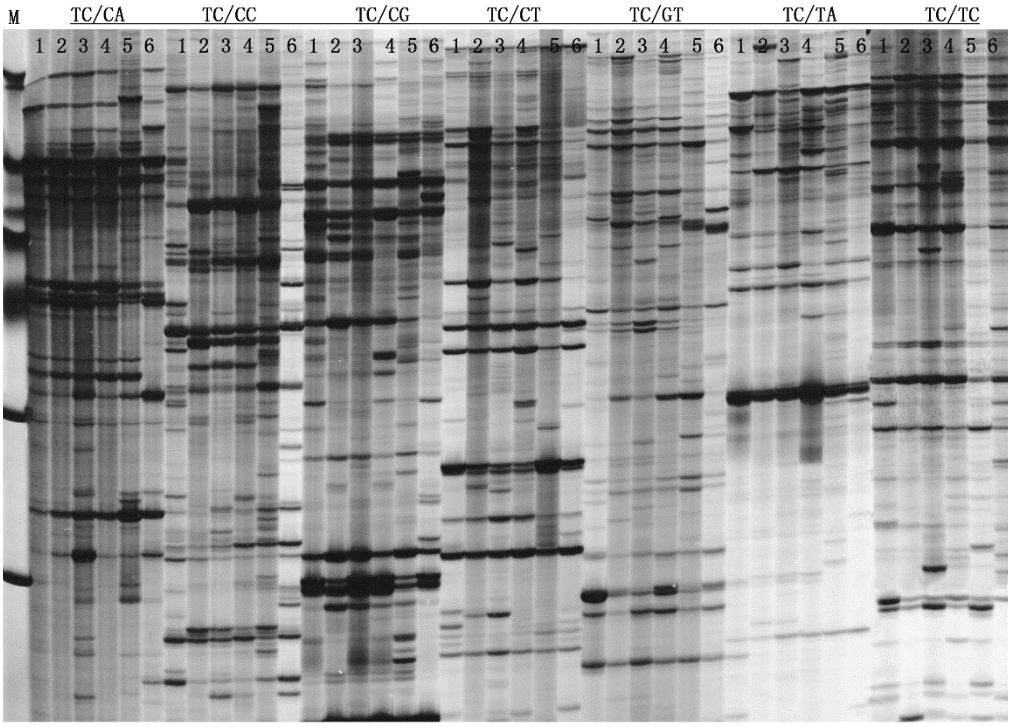

Figure S1 A representative picture of a silver-stained cDNA-AFLP gel showing the transcripts in tangor leaves in response to drought stress using one *Ase* I selective primer (*Ase* I-TC) and seven *Taq* I selective primers (*Taq*I-CA, CC, CG, CT, GT, TA and TC). 1~6: 0, 3, 5, 8, 11 and 14 day after drought treatment, respectively; M: molecular weight marker. Lane 6 was only the reference and not used for results analysis.

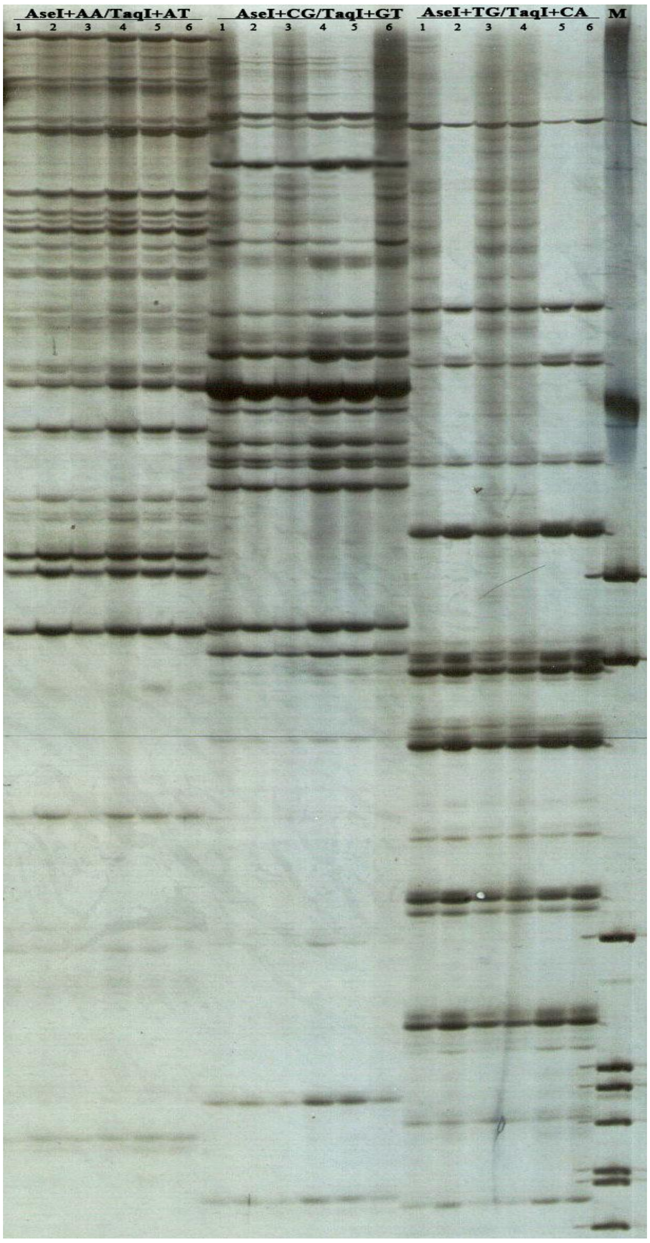

Figure S2 A cDNA-AFLP profile of control plants across the drought period. Three pair of primer combinations (*Ase*I+AA/*Taq*I+AT, *Ase*I+CG/*Taq*I+GT and *Ase*I+TG/*Taq*I+CA) were randomly selected to perform the cDNA-AFLP procedure. 1~6: 0, 3, 5, 8, 11 and 14 day on control plants, respectively; M: molecular weight marker. Lane 6 was only the reference and not used for results analysis.
